# 2435: Accuracies of using Her2 for prognosis of breast cancer recurrence in Life After Cancer Epidemiology (LACE) Study

**DOI:** 10.1017/cts.2017.106

**Published:** 2018-05-10

**Authors:** Nan Hu

**Affiliations:** The University of Utah School of Medicine, Salt Lake City, UT, USA

## Abstract

OBJECTIVES/SPECIFIC AIMS: The goal of the study is to evaluate the prognostic importance and accuracies of a biomarker, human epidermal receptor 2 (Her2), for breast cancer recurrence in a cohort study, namely Lifetime after Cancer Epidemiology (LACE). We specifically interested in the role that Her2 plays in prognosis of breast tumor recurrence for women after a previously diagnosed and treated breast cancer. METHODS/STUDY POPULATION: The study cohort includes 2267 women enrolled in LACE who had previously diagnosed breast cancer. Patients were enrolled from each of the 2 LACE registries in California and Utah. The main endpoint of the study is the right-censored time to breast cancer recurrence. Patients’ enrollments were, on average, 2 years after diagnosis of the first breast cancer. The patients’ characteristics at baseline were obtained through self-administered questionnaires. Cox proportional hazard model with time-varying covariates was used to relate the Her2 status (Her2+ and Her2−) to the primary end point (time to breast cancer recurrence). Hazard ratios (HRs) and their 95% confidence interval comparing Her2+ and Her2− arms were estimated. Time-dependent sensitivity and specificity were used to investigate the performance of using Her2 for classifying patients into high and low risk (Her2 + is classified as hi risk and Her− as low risk) of future breast cancer recurrence at time points after baseline. The time-dependent sensitivity was calculated as the proportion of patients being classified as high risk of recurrence who had breast cancer recurrence before a series of pre-specified time points after baseline, and the time-dependent specificity was calculated as the proportion of subjects being classified as low risk of recurrence who did not have breast cancer recurrence at the same time points. RESULTS/ANTICIPATED RESULTS: The average patient follow-up time was 9.8 years, and 18% of the women got positive Her2 test results at baseline. Among 2267 patients in the study cohort, 2031 had records on their Her2 status, among whom 326 (16.1%) patients were Her2+ and 1705 (83.9%) were Her2−. The mean tumor size among the 2031 patient was 2.10±1.22 cm. A majority of the patients (78.9%) were White. Over one-half of these patients were neither current nor past smokers. Only 3% of the patient had a baseline stage IIIA or higher. About 49% of the patients underwent a mastectomy. Radiation therapy was used by 63.5% of the patients, and Tamoxifen users accounted for 78% of the study cohort. We found a statistically significant association between Her2 and breast cancer recurrence (HR=1.33, log-ran *p*-value=0.006). However, the HRs of breast cancer recurrence comparing Her2+ and Her2− patients decreased over time. We also investigate the effect of combined Her2, estrogen (ER), and progesterone (PR) on breast cancer recurrence and found that patients with Her2+/ER+/PR− had the highest risk of breast cancer recurrence. The hazard of recurrence for this group of patients was 85% higher than patients with Her2−/ER+/PR+. We also investigate the prognostic accuracies of Her2 in terms of time-dependent sensitivity and specificity. Using Her2 as the prognostic biomarker resulted in a specificity consistently over 80% from baseline up until 15 years post-baseline. The time-dependent sensitivity of Her2 was above 90% between baseline and 1.5 years. Then, the sensitivity dropped gradually to 40% from 1.5 years to 3 years post-baseline. For prognosis of breast cancer over 3 years from baseline, the sensitivity was between 30% and 40%. DISCUSSION/SIGNIFICANCE OF IMPACT: As a single biomarker and risk factor, Her2 was statistically significantly associated with the recurrence of breast cancer among patients in the LACE cohort. A composite biomarker by combining Her2, ER, and PR status was also significantly associated with the breast cancer recurrence. However, the HRs of breast cancer recurrence comparing Her2+ and Her2− patients decreased over time, implying that the Her2 status had a high impact on early recurrent breast tumors. Single biomarkers, usually, have very limited ability for prognosis of future events. However, we found that using HER2 as a single biomarker can give a relatively larger specificity consistently over 15 years of the study period. The sensitivity of Her2 is high for detecting early breast cancer recurrence. However, after 2.5 years from baseline, using Her2 for breast cancer recurrence detection is not reliable. Due to the relatively high accuracies of using Her2 status for prognosis of breast cancer recurrence, we conclude that Her2 should be considered in clinical studies related to prognosis of breast cancer recurrence. Future studies will investigate if prognostic accuracies can be improved by combining Her2 with baseline clinical risk factors such as age, tumor size and lymph nodes. In conclusion, our study has the clinical impact on prognosis (or early detection) of breast cancer recurrence among women with previously diagnosed and treated breast cancers.

